# Safety of TNF-α inhibitors: A real-world study based on the US FDA Adverse Event Reporting System Database

**DOI:** 10.1097/MD.0000000000039012

**Published:** 2024-07-19

**Authors:** Bohui Zheng, Manting Liu, Dandan Dai, Yifan Shang, Xiangyun Dou, Bingshuo Liu, Zilan Zhong, Shulan Huang, Dongqiang Luo

**Affiliations:** a Clinical Medical College of Acupuncture-Moxibustion and Rehabilitation, Guangzhou University of Chinese Medicine, Guangzhou, China; b Clifford Hospital, Guangzhou University of Chinese Medicine, Guangzhou, China; c The First Clinical College, Guangzhou University of Chinese Medicine, Guangzhou, China; d College of Traditional Chinese Medicine, Guangzhou University of Chinese Medicine, Guangzhou, China; e The Fifth Clinical College, Guangzhou University of Chinese Medicine, Guangzhou, China; f General Hospital of Guangzhou Military Command of PLA, Guangzhou, China.

**Keywords:** adverse drug events, FAERS, immunity, TNF-α inhibitors

## Abstract

As a common treatment for rheumatoid arthritis (RA), the adverse reactions of TNF-α inhibitors (TNFis) in practical application have garnered attention. This study aims to investigate the adverse drug events (ADEs) associated with TNFi in RA patients as reported in the FDA Adverse Event Reporting System, to offer insights for clinical use. Cases related to RA and primarily involving TNFi were extracted from the FDA Adverse Event Reporting System database and compared by gender stratification. Screening was conducted based on reporting odds ratio and information component to identify positive ADEs for different TNFis and evaluate common and unique ADEs among various TNFis. There are 4 common ADEs among TNFis, including pulmonary tuberculosis, infection, hypersensitivity, and herpes zoster, as described in the package inserts. However, each TNFi has unique positive ADEs. Adalimumab has 63 unique positive ADEs, including lower respiratory tract inflammation, systemic lupus erythematosus rash, vascular dementia, ovarian neoplasm, adhesion, sarcoma, coccidioidomycosis, etc. Golimumab has 6 unique positive ADEs, including pneumonia cryptococcal, device deployment issue, pneumonia bacterial, polyneuropathy, device malfunction, device issue, etc; certolizumab has 24 unique positive ADEs, including maternal exposure before pregnancy, premature rupture of membranes, exposure via breast milk, staphylococcal sepsis, erysipelas, low birth weight baby, herpes virus infection, premature delivery, etc; etanercept has 180 unique positive ADEs, including joint destruction, chondrolysis, finger deformity, ankle deformity, joint warmth, etc; infliximab has 60 unique positive ADEs, including Hodgkin’s disease, metastatic neoplasm, non-Hodgkin’s Lymphoma, etc. Although the aforementioned 5 TNFis share common ADEs such as herpes zoster, clinicians must exercise caution when selecting specific medications, especially for RA patients concurrently suffering from malignancies. The analysis indicates that infliximab is associated with 60 unique positive ADEs, including Hodgkin’s disease, metastatic neoplasm, and non-Hodgkin’s lymphoma; therefore, these patients should use infliximab with greater caution. Similarly, certolizumab should be used with increased caution in pregnant and postpartum women.

## 1. Introduction

Autoimmune diseases are commonly managed with potent immunotherapies such as tumor necrosis factor (TNF) inhibitors (TNFis), and to date, the US Food and Drug Administration (FDA) has approved 5 TNF inhibitors: adalimumab, golimumab, certolizumab, etanercept, and infliximab: for the treatment of these conditions.^[1]^ TNFis have been extensively utilized in a variety of autoimmune and inflammatory diseases, such as rheumatoid arthritis (RA), psoriatic arthritis, ankylosing spondylitis, juvenile idiopathic arthritis, and inflammatory bowel diseases (IBD), which include Crohn’s disease and ulcerative colitis.^[2–4]^ While the widespread use of TNFis in patients with RA is notable, it has also raised concerns regarding potential adverse reactions.

Despite the fact that drug leaflets and FDA warnings provide important safety information, it is still necessary to continue exploring potential adverse reactions of drugs.^[5]^ Lasser et al^[6]^ analyzed the timing patterns of FDA-added “black box warnings” and market withdrawals, discovering that many serious adverse reactions were not identified and warned about until years after the drugs were marketed. Berlin et al ^[7]^ (2008) pointed out that rare adverse events (AEs) may only be discovered post-marketing, suggesting continuous monitoring and evaluation of AEs throughout the entire lifecycle of drug development. According to summaries in product safety descriptions, the risk of AEs associated with TNF inhibitors is relatively low.^[8–10]^ However, severe adverse reactions to anti-TNFα agents have been reported in clinical trials and post-marketing studies for approved indications.^[11–13]^ These adverse effects compromise the immune system, weakening the patient’s ability to resist infections and cancers.^[14]^ Life-threatening and fatal infections, including sepsis, as well as various malignancies, have been documented. There have also been reports of cardiovascular, hematological, neurological, and autoimmune reactions.^[15–17]^ The British Society for Rheumatology believes this is a rapidly changing field, with new adverse reaction data emerging every month, and based on the 2001 and 2005 British Society for Rheumatology guidelines and the latest literature search data, they propose “if malignancy is clinically suspected, it should be investigated, and if confirmed, anti-TNF drugs should be discontinued.”^[18]^ However, clinicians should update their knowledge of potential ADEs in real-time to facilitate better clinical application.

Identifying potential adverse drug events (ADEs) early in the drug development process and proactively predicting their occurrence in humans is one of the paramount responsibilities of the pharmaceutical industry and regulatory authorities, and it is also a concern for patients. However, due to limited participant numbers and exposure duration, identifying all ADEs in clinical trials is exceptionally challenging, making signal detection one of the critical methodologies for uncovering any new AEs. The World Health Organization defines a signal as “reported information on a possible causal relationship between an AE and a drug, where the relationship is either unknown or incompletely documented.” This study aims to employ disproportionality analysis of the FDA Adverse Event Reporting System (FAERS) database to generate signals concerning previously unreported adverse drug reactions associated with TNFis.^[19]^

## 2. Materials and methods

### 2.1. Data sources

Spontaneous reporting systems are a vital source for monitoring adverse drug reactions, and the publicly accessible FAERS is one of the critical databases of such systems. FAERS compiles cases of adverse reactions reported to the FDA by healthcare professionals, pharmaceutical manufacturers, and consumers from various countries. Each report gathers a patient’s clinical characteristics, including age, gender, country, indications for use, types of adverse reactions, date of medication initiation, and the time of the AE. OpenVigil FDA is an analytical tool that uses an Application Programming Interface to search the FAERS database, enabling researchers to access and utilize data from the FAERS more efficiently.^[20,21]^ Each event is coded using the Medical Dictionary for Regulatory Activities standardized terminology.^[22]^ Data were collected using the OpenVigil FDA tool imported into R software version 4.2.3 for data cleansing, removal of duplicate report IDs, and subsequent analysis.

### 2.2. Exposure definition

Infliximab, etanercept, and adalimumab were approved by the FDA in 1998, 1998, and 2002 respectively for the treatment of RA. This study extracts data from the FAERS database starting from the earliest available year of 2004 and extends the study window to the present, allowing us to capture safety information related to the use of these drugs. AE data for TNF inhibitors were retrieved from the FAERS database from the first quarter of 2004 to the third quarter of 2023. The TNFis included in this study were adalimumab, golimumab, certolizumab, etanercept, and infliximab. To include as much data as possible, this study queried the Pharnexcloud database (https://www.pharnexcloud.com/) for various countries’ drug and commercial names of the TNFis above. After deduplication, these terms were used as search keywords for TNFis, retaining only those reports with RA as the unique diagnosis. Reports where TNFis were identified as the primary suspect (PS) were extracted. In calculating the time to onset, reports with an event occurring more than 720 days after were excluded.^[23]^ Only those provided by physicians, pharmacists, and other health specialists were included in this study to ensure the credibility of the reports.

### 2.3. Disproportionality analysis and statistical analysis

Data were analyzed using R software version 4.2.3 for disproportionality analysis, with a *P*-value < .05 considered statistically significant, and the Bonferroni correction was applied for multiple testing adjustments. The Reporting Odds Ratio (ROR) and the quantity of information component (IC) using the Bayesian Confidence Propagation Neural Network were used for disproportionality analysis. Signals for disproportionate reporting were required to meet the following criteria^[24]^: (A) ROR025 (the lower limit of the 95% confidence interval for ROR) >1 and the AE count > 3; (B) IC025 (the lower limit of the 95% credible interval for IC) > 0. Sensitivity analyses were conducted through sex stratification to evaluate the positive signals for ADEs in different genders. To further identify common positive ADEs across TNFis and those unique to each drug, data were visualized and analyzed using UpSet plots.

## 3. Results

### 3.1. Characteristics of case reports

In this study, the FAERS database included 16,964,230 drug event combinations reported from the first quarter of 2004 to the third quarter of 2023. There were 3441 drug event combinations with adalimumab as the primary suspect (PS), 918 with golimumab as PS, 1190 with certolizumab as PS, 15,730 with etanercept as PS, and 1430 with Infliximab as PS. Reports related to TNFis primarily featured a higher proportion of females; most patients weighed between 50 and 100 kg, and the majority were aged 18 to 64. The top 5 reporting countries were the United States, Japan, Canada, the United Kingdom, France, and Germany (Table 1).

**Table 1:** General information on the reports included in the study.

Variate		Adalimumab	Golimumab	Certolizumab	Etanercept	Infliximab
		(N = 3441)	(N = 918)	(N = 1190)	(N = 15,730)	(N = 1432)
SEX	Female	2456 (71.4%)	645 (70.3%)	914 (76.8%)	12,690 (80.7%)	839 (58.6%)
	Male	649 (18.9%)	192 (20.9%)	243 (20.4%)	2806 (17.8%)	298 (20.8%)
	Uncertain	336 (9.8%)	81 (8.8%)	33 (2.8%)	234 (1.5%)	295 (20.6%)
Weight (kg)	<50	62 (1.8%)	59 (6.4%)	50 (4.2%)	108 (0.7%)	57 (4.0%)
	50–100	778 (22.6%)	175 (19.1%)	291 (24.5%)	1017 (6.5%)	364 (25.4%)
	>100	97 (2.8%)	14 (1.5%)	27 (2.3%)	157 (1.0%)	37 (2.6%)
	Uncertain	2504 (72.8%)	670 (73.0%)	822 (69.1%)	14,448 (91.9%)	974 (68.0%)
Age (years)	≤17	6 (0.2%)	1 (0.1%)	13 (1.1%)	53 (0.3%)	2 (0.1%)
	18–64	1160 (33.7%)	287 (31.3%)	592 (49.7%)	9862 (62.7%)	471 (32.9%)
	65–85	690 (20.1%)	249 (27.1%)	242 (20.3%)	2833 (18.0%)	370 (25.8%)
	≥86	24 (0.7%)	13 (1.4%)	3 (0.3%)	56 (0.4%)	5 (0.3%)
	Uncertain	1561 (45.4%)	368 (40.1%)	340 (28.6%)	2926 (18.6%)	584 (40.8%)
Reporter	Physician	2461 (71.5%)	597 (65.0%)	658 (55.3%)	12,740 (81.0%)	901 (62.9%)
	Other health specialists	617 (17.9%)	211 (23.0%)	294 (24.7%)	2470 (15.7%)	443 (30.9%)
	Pharmacist	363 (10.5%)	110 (12.0%)	238 (20.0%)	520 (3.3%)	88 (6.1%)
Top 5 report region		United States (38.2%)	United States (32.8%)	United States (34.5%)	United States (88.4%)	United States (29.3%)
		Canada (4.4%)	Japan (23.7%)	Japan (11.2%)	Japan (2.4%)	Canada (8.2%)
		United Kingdom (2.0%)	Germany (9.9%)	Germany (7.6%)	Canada (1.3%)	Japan (5.6%)
		Germany (3.6%)	Colombia (6.2%)	France (6.1%)	Taiwan (0.7%)	United Kingdom (3.4%)
		Czech Republic (0.9%)	Canada (5.0%)	Canada (4.9%)	France (0.7%)	France (3.3%)

**Figure 1.**
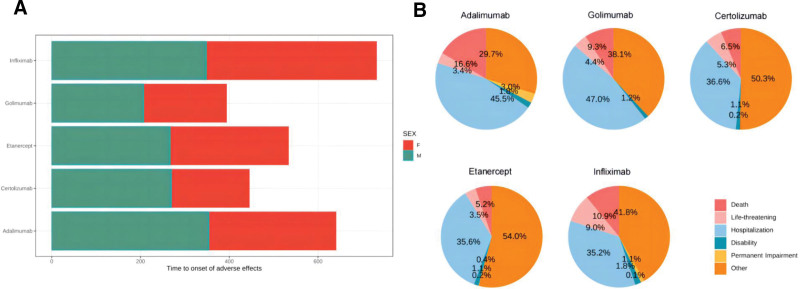
(A) Comparison of time to onset of ADE in TNFis. (B) Comparison of ADE outcomes in TNFis. ADE = adverse drug event, TNFis = tumor necrosis factor inhibitors.

Within the TNFis, women experienced a shorter time to onset of effects, and overall, golimumab, certolizumab, and etanercept had shorter times to onset (Fig. 1A); the proportion of deaths potentially due to TNFis was not insubstantial (16.6%, 9.3%, 6.5%, 5.2%, and 10.9%), with a high percentage of these events leading to hospitalization, all exceeding 35% (45.5%, 47.0%, 36.6%, 35.6%, and 35.2%) (Fig. 1B).

### 3.2. Detection of positive signals

**Figure 2.**
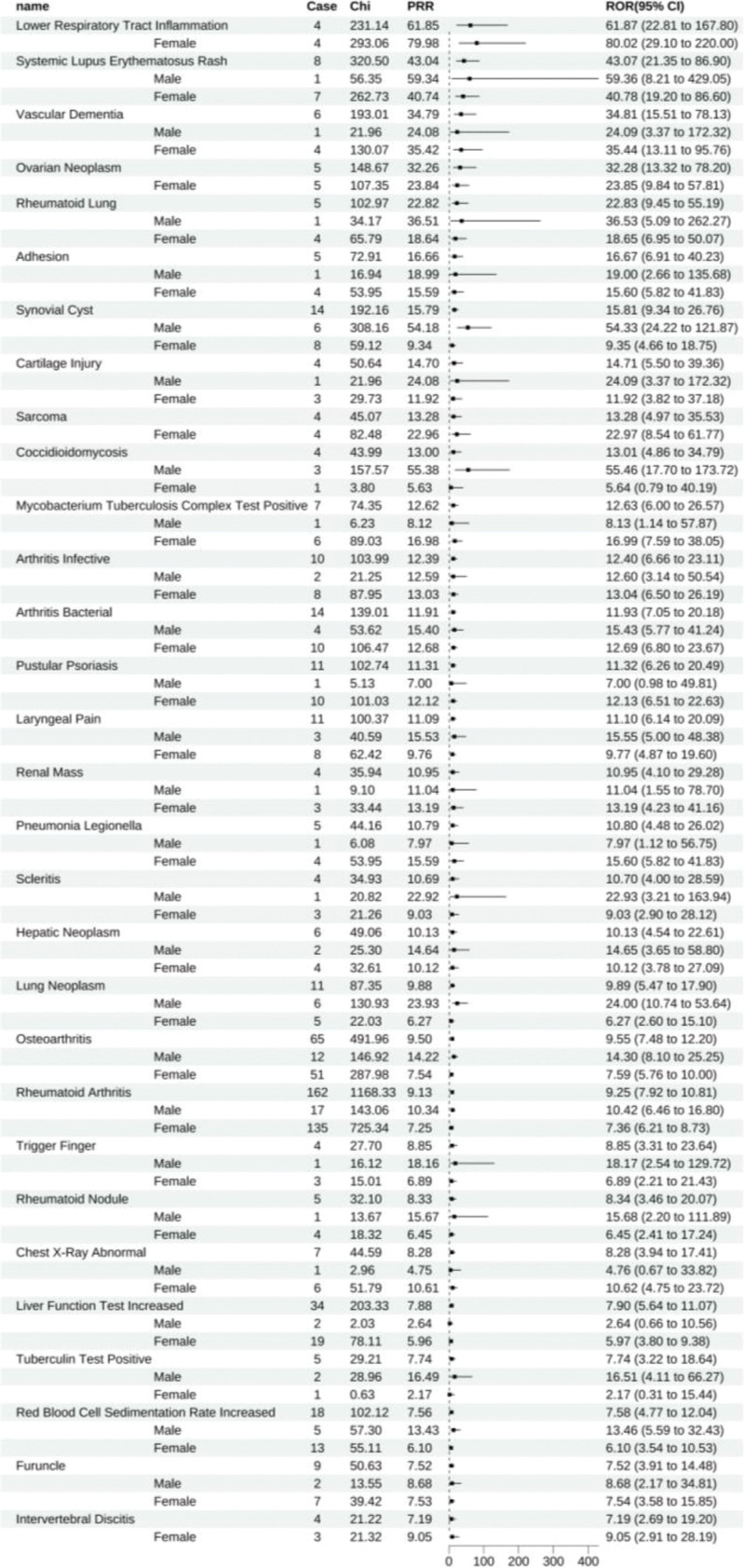
Adalimumab-positive ADE by ROR Top 30 Forest Graph. ADE = adverse drug event, ROR = The Reporting Odds Ratio.

Upon satisfying the criteria wherein the lower bound of the 95% confidence interval for the reporting odds ratio (ROR025) exceeds 1, the number of AEs is more excellent than 3. The lower bound of the 95% confidence interval for the IC (IC025) surpasses zero, signaling a positive threshold. A disproportionality analysis revealed that adalimumab exhibited 178 positive signals. When ranked by ROR, the top ten ADEs were inflammation of lower respiratory tract inflammation (All: 61.87 [22.81–167.80], female: 80.02 [29.10–220.00]), systemic lupus erythematosus rash (All: 43.07 [21.35–86.90], male: 59.36 [8.21–429.05]), female: 40.78 [19.20–86.60]), vascular dementia (All: 34.82 [15.51–78.13], male: 24.09 [3.37–172.32], female: 35.44 [13.11–95.76]), ovarian neoplasm (All: 32.28 [13.32–78.20], female: 23.85 [9.84–57.81]), rheumatoid lung (All: 22.83 [9.45–55.19], male: 36.53 [5.09–262.27], female: 18.65 [6.95–50.07]), adhesion (All: 16.67 [6.91–40.23], male: 19.00 [2.66–135.68], female: 15.60 [5.82–41.83]), synovial cyst (All: 15.81 [9.34–26.76], male: 54.33 [24.22–121.87], female: 9.35 [4.66–18.75]), cartilage injury (All: 14.71 [5.50–39.36], male: 24.09 [3.37–172.32], female: 11.92 [3.82–37.18]), sarcoma (All: 13.28 [4.97–35.53], female: 22.97 [8.54–61.77]), coccidioidomycosis (All: 13.01 [4.86–34.79], male: 55.46 [17.70–173.32], female: 5.64 [0.79–40.19]), stratification by gender within these ten prevalent ADEs unearthed that male representation was disproportionately low, with no statistical significance, in systemic lupus erythematosus rash, vascular dementia, rheumatoid lung, adhesions, and cartilage injury. In contrast, the remaining ADEs manifested as positive signals in both males and females (Fig. 2).

Golimumab was associated with 41 positive signals, and the top ten ADEs determined by ROR were pneumonia cryptococcal (All: 91.96 [34.33–246.37], male: 14.09 [1.97–100.46], female: 15.70 [3.89–63.32]), pulmonary tuberculosis (All: 46.43 [26.28–82.01], male: 10.11 [3.79–27.00], female: 1.19 [0.17–8.47]), arthritis bacterial (All: 35.68 [16.96–75.05], male: 15.43 [5.77–41.24], female: 12.69 [6.80–23.67]), disseminated tuberculosis (All: 32.74 [13.59–78.86], female: 7.49 [2.80–20.03]), issues with device deployment (All: 31.66 [15.79–63.48]), pneumonia bacterial (All: 22.07 [11.01–44.23], male: 3.54 [0.88–14.16], female: 1.74 [0.43–6.97]), pyelonephritis (All: 17.13 [8.15–36.00], female: 1.33 [0.43–4.12]), basal cell carcinoma (All: 13.16 [6.83–25.35], male: 1.07 [0.15–7.59], female: 2.25 [1.01–5.01]), lymphoma (All: 12.72 [6.35–25.50], male: 5.81 [2.41–13.99], female: 4.26 [2.21–8.20]), and kidney infection (All: 12.45 [5.17–29.97], female: 2.43 [1.09–5.42]).

**Figure 3.**
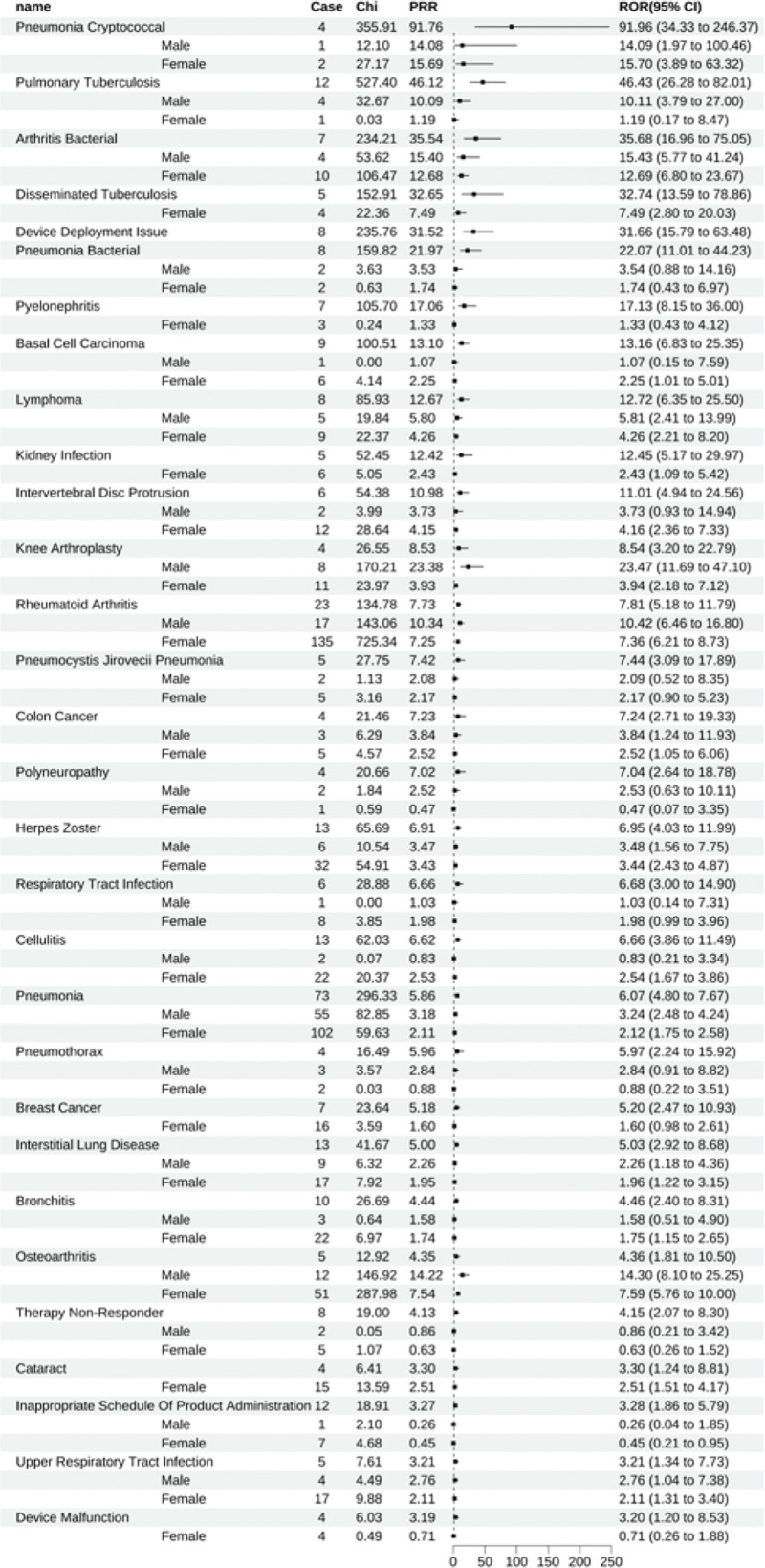
Golimumab-positive ADE by ROR Top 30 Forest Graph. ADE = adverse drug event, ROR = The Reporting Odds Ratio.

Gender stratification within these 10 ADEs disclosed that aside from pneumonia cryptococcal (All: 91.96 [34.33–246.37], male: 14.09 [1.97–100.46], female: 15.70 [3.89–63.32]), pulmonary tuberculosis (All: 46.43 [26.28–82.01], male: 10.11 [3.79–27.00], female: 1.19 [0.17–8.47]), pneumonia bacterial (All: 22.07 [11.01–44.23], male: 3.54 [0.88–14.16], female: 1.74 [0.43–6.97]), and basal cell carcinoma (All: 13.16 [6.83–25.35], male: 1.07 [0.15–7.59], female: 2.25 [1.01–5.01]), which reported scant numbers (fewer than 3 cases) and thus lacked statistical significance, the rest of the ADEs were detected as positive signals across both genders (Fig. 3).

**Figure 4.**
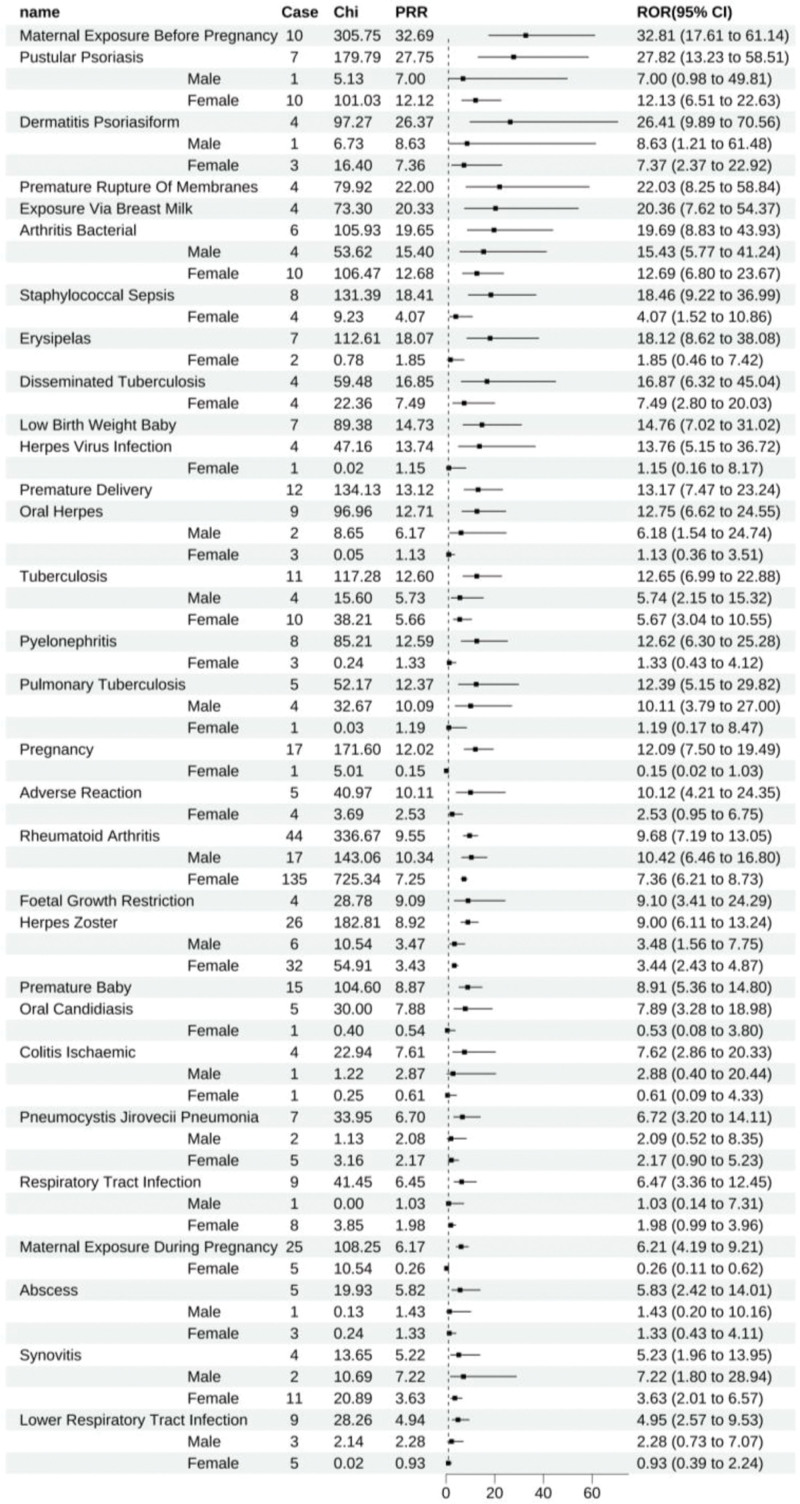
Certolizumab-positive ADE by ROR Top 30 Forest Graph. ADE = adverse drug event, ROR = The Reporting Odds Ratio.

Certolizumab pegol was linked to 70 positive signals, and the top 10 ADEs, in order of ROR, included maternal exposure prior to pregnancy (All: 32.81 [17.61–61.14]), pustular psoriasis (All: 27.82 [13.23–58.51], male: 7.00 [0.98–49.81], female: 12.13 [6.51–22.63]), psoriasiform dermatitis (All: 26.41 [9.89–70.56], male: 8.63 [1.21–61.48], female: 7.37 [2.37–22.92]), premature rupture of membranes (All: 22.03 [8.25–58.84]), exposure through breast milk (All: 20.36 [7.62–54.37]), bacterial arthritis (All: 19.69 [8.83–43.93], male: 15.43 [5.77–41.24], female: 12.69 [6.80–23.67]), staphylococcal sepsis (All: 18.46 [9.22–36.99], female: 4.07 [1.52–10.86]), erysipelas (All: 18.12 [8.62–38.08], female: 1.85 [0.46–7.42]), disseminated tuberculosis (All: 16.87 [6.32–45.04], female: 7.49 [2.80–20.03]), and low birth weight in infants (All: 14.76 [7.02–31.02]). Gender-based analysis of these 10 ADEs indicated that due to the paucity of reports for pustular psoriasis, psoriasiform dermatitis, and erysipelas (fewer than 3 cases), gender stratification was not statistically significant. In contrast, other ADEs demonstrated positive signals upon stratification (Fig. 4).

**Figure 5.**
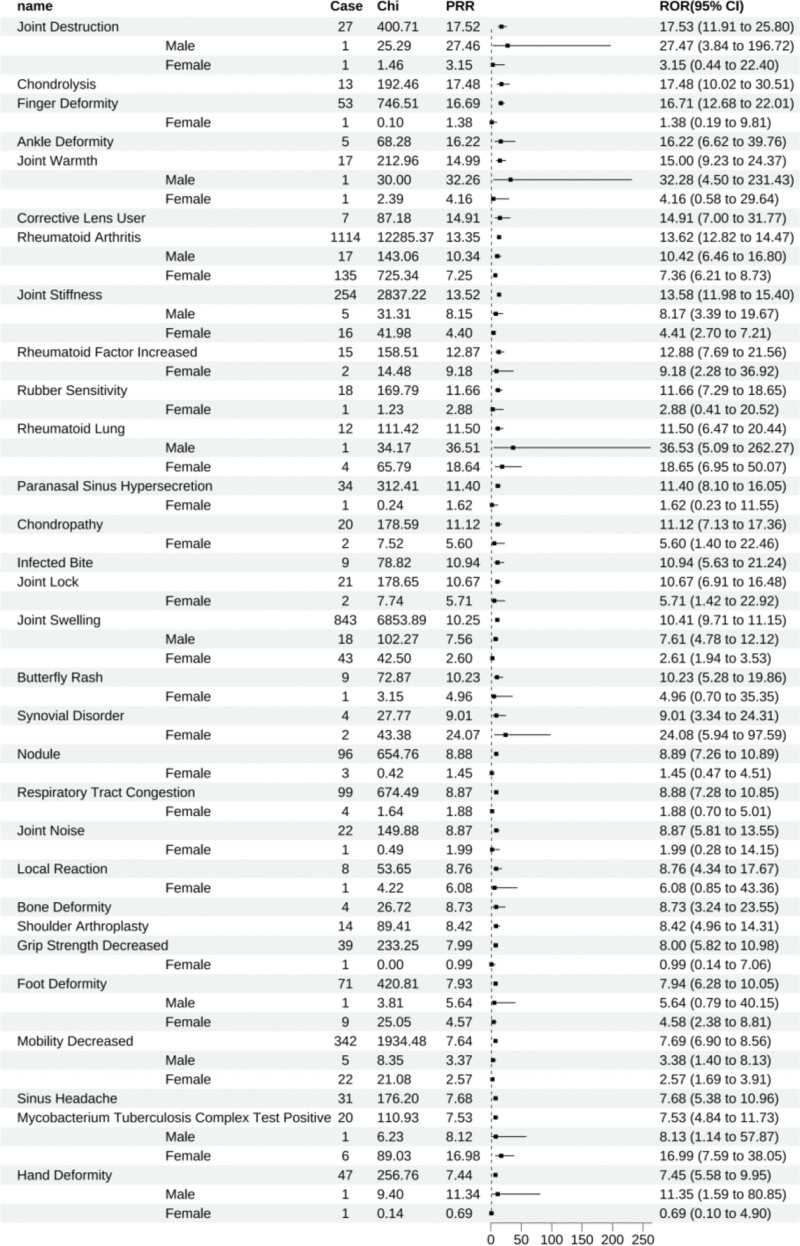
Etanercept-positive ADE by ROR Top 30 Forest Graph. ADE = adverse drug event, ROR = The Reporting Odds Ratio.

Etanercept disclosed 299 positive signals. Ranking these by ROR, the primary ADEs encompassed joint destruction (All: 17.53 [11.91–25.80], male: 27.47 [3.84–196.72], female: 3.15 [0.44–22.40]), chondrolysis (All: 17.48 [10.02–30.51]), finger deformity (All: 16.71 [12.68–22.01], female: 1.38 [0.19–9.81]), ankle deformity (All: 16.22 [6.62–39.76]), joint warmth (All: 15.00 [9.23–24.37], male: 32.28 [4.50–231.43], female: 4.16 [0.58–29.64]), those who require corrective lenses (All: 14.91 [7.00–31.77]), RA (All: 13.62 [12.82–14.47], male: 10.42 [6.46–16.80], female: 7.36 [6.21–8.73]), joint stiffness (All: 13.58 [11.98–15.40], male: 8.17 [3.39–19.67], female: 4.41 [2.70–7.21]), increased rheumatoid factor (All: 12.88 [7.69–21.56], female: 9.18 [2.28–36.92]), and sensitivity to rubber (All: 11.66 [7.29–18.65], female: 2.88 [0.41–20.52]). Gender stratification among these principal ADEs revealed an insufficient number of gender-specific reports for joint destruction, finger deformity, joint warmth, and sensitivity to rubber, rendering the gender stratification statistically insignificant (Fig. 5).

**Figure 6.**
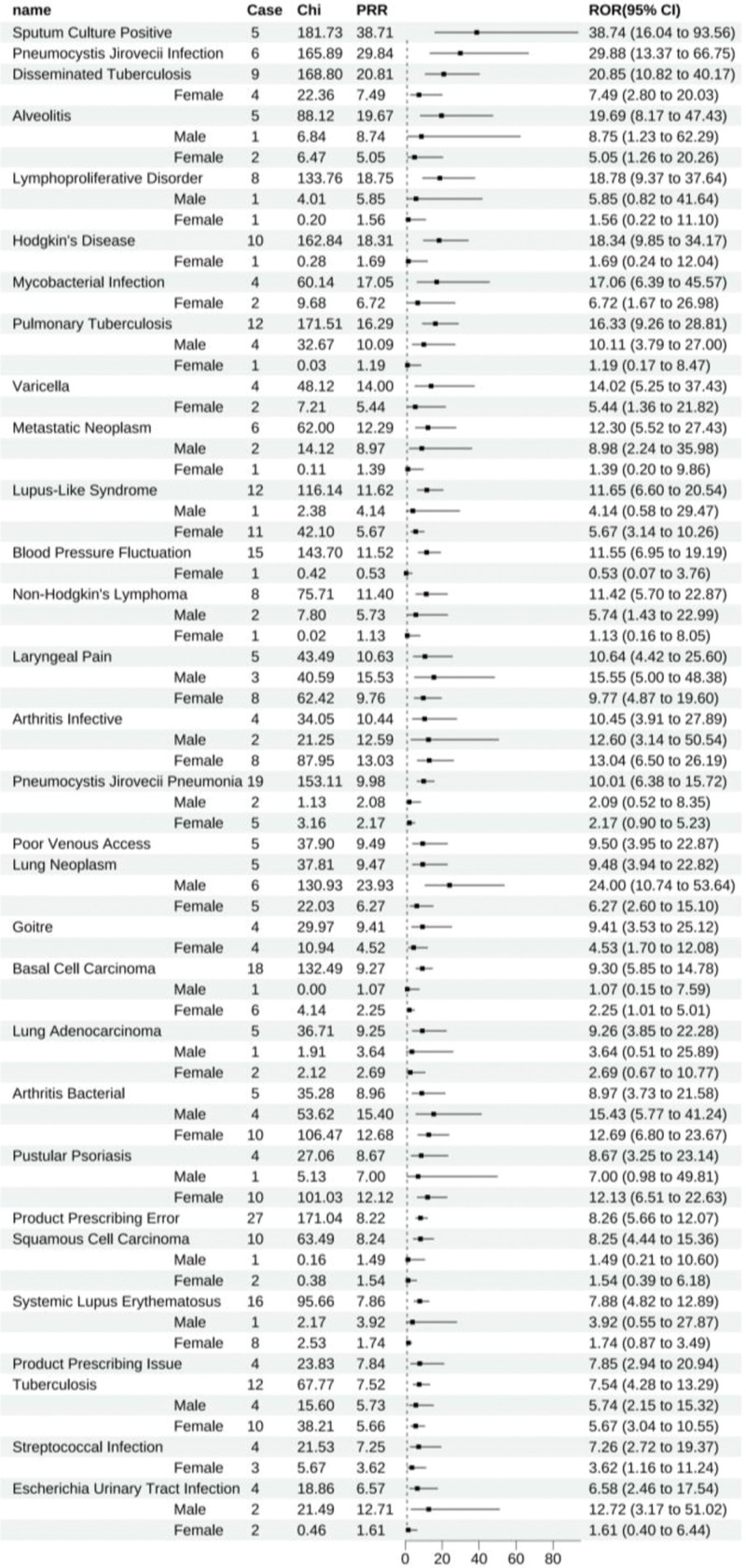
Infliximab-positive ADE by ROR Top 30 Forest Graph. ADE = adverse drug event, ROR = The Reporting Odds Ratio.

Infliximab revealed 114 positive signals, with the ten foremost ADEs being sputum culture positivity (All: 38.74 [16.04–93.56]), *Pneumocystis jirovecii* infection (All: 29.88 [13.37–66.75]), disseminated tuberculosis (All: 20.85 [10.82–40.17], female: 7.49 [2.80–20.03]), alveolitis (All: 19.69 [8.17–47.43], male: 8.75 [1.23–62.29], female: 5.05 [1.26–20.26]), lymphoproliferative disorder (All: 18.78 [9.37–37.64], male: 5.85 [0.82–41.64], female: 1.56 [0.22–11.10]), Hodgkin’s disease (All: 18.34 [9.85–34.17], female: 1.69 [0.24–12.04]), mycobacterial infection (All: 17.06 [6.39–45.57], female: 6.72 [1.67–26.98]), pulmonary tuberculosis (All: 16.33 [9.26–28.81], male: 10.11 [3.79–27.00], female: 1.19 [0.17–8.47]), varicella (All: 14.02 [5.25–37.43], female: 5.44 [1.36–21.82]), and metastatic neoplasm (All: 12.30 [5.52–27.43], male: 8.98 [2.24–35.98], female: 1.39 [0.20–9.86]). Within these leading ADEs, gender stratification demonstrated a lack of valid gender-specific reports for conditions such as alveolitis, lymphoproliferative disorder, and Hodgkin’s disease, leading to a lack of statistical significance in gender stratification (Fig. 6).

### 3.3. Jointly shared positive ADE detection

**Figure 7.**
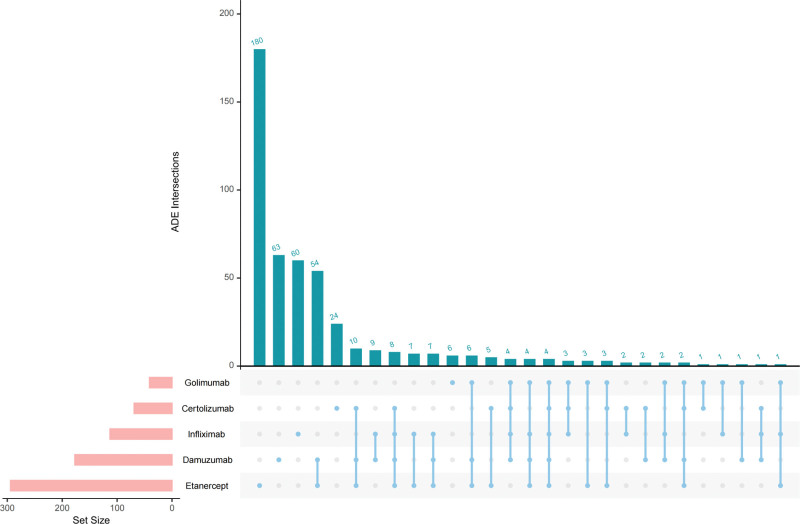
TNFis positive ADE intersection Upset plot. ADE = adverse drug event, TNFis = tumor necrosis factor inhibitors.

**Figure 8.**
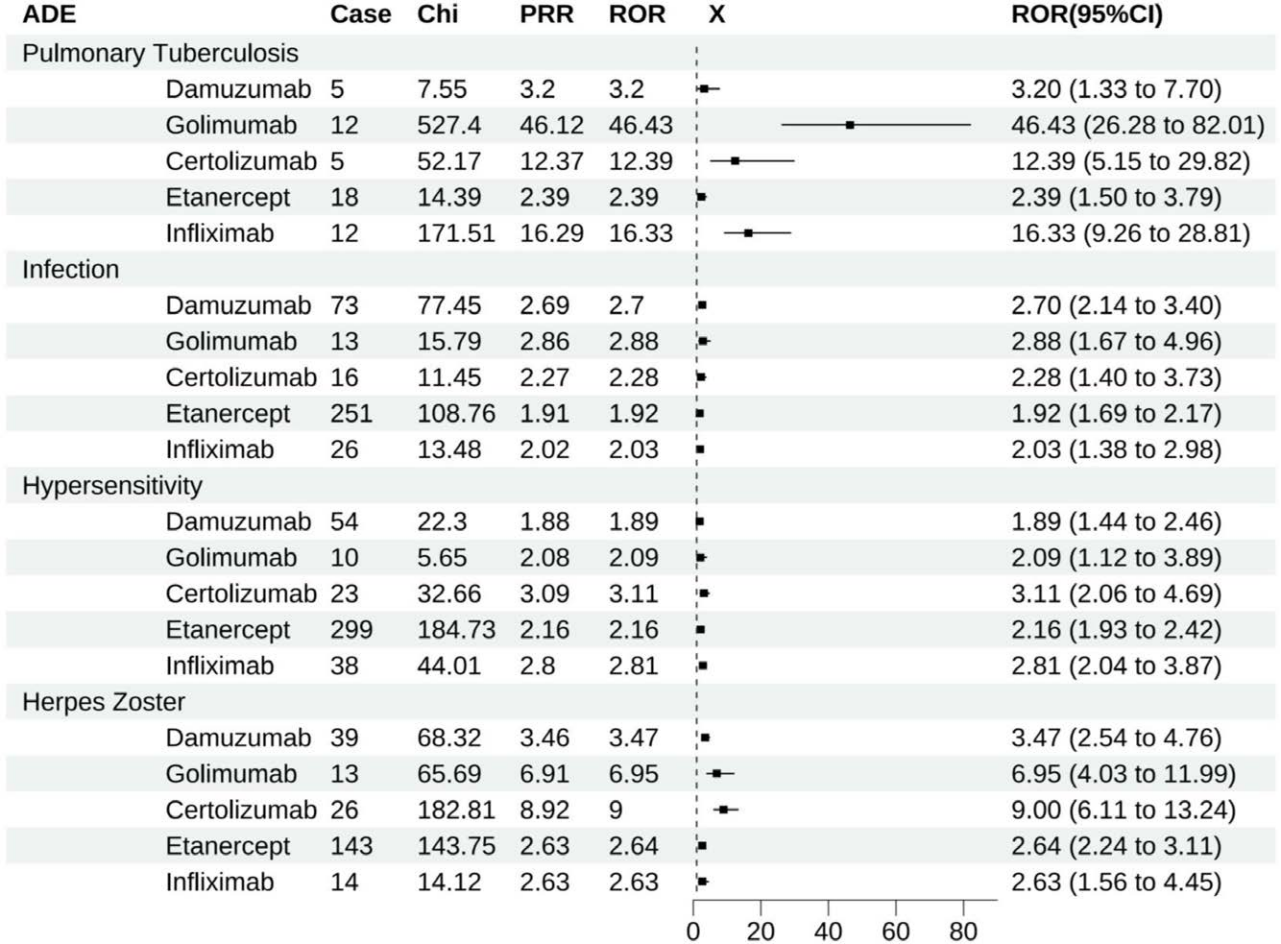
Positive ADEs shared by TNFis. ADEs = adverse drug events, TNFis = tumor necrosis factor inhibitors.

Through intersection analysis (Fig. 7), which identified 4 positive ADEs common to all 5 types of TNFis, namely pulmonary tuberculosis, infection, hypersensitivity, and herpes zoster (Fig. 8).

### 3.4. Unique positive ADE detection

**Figure 9.**
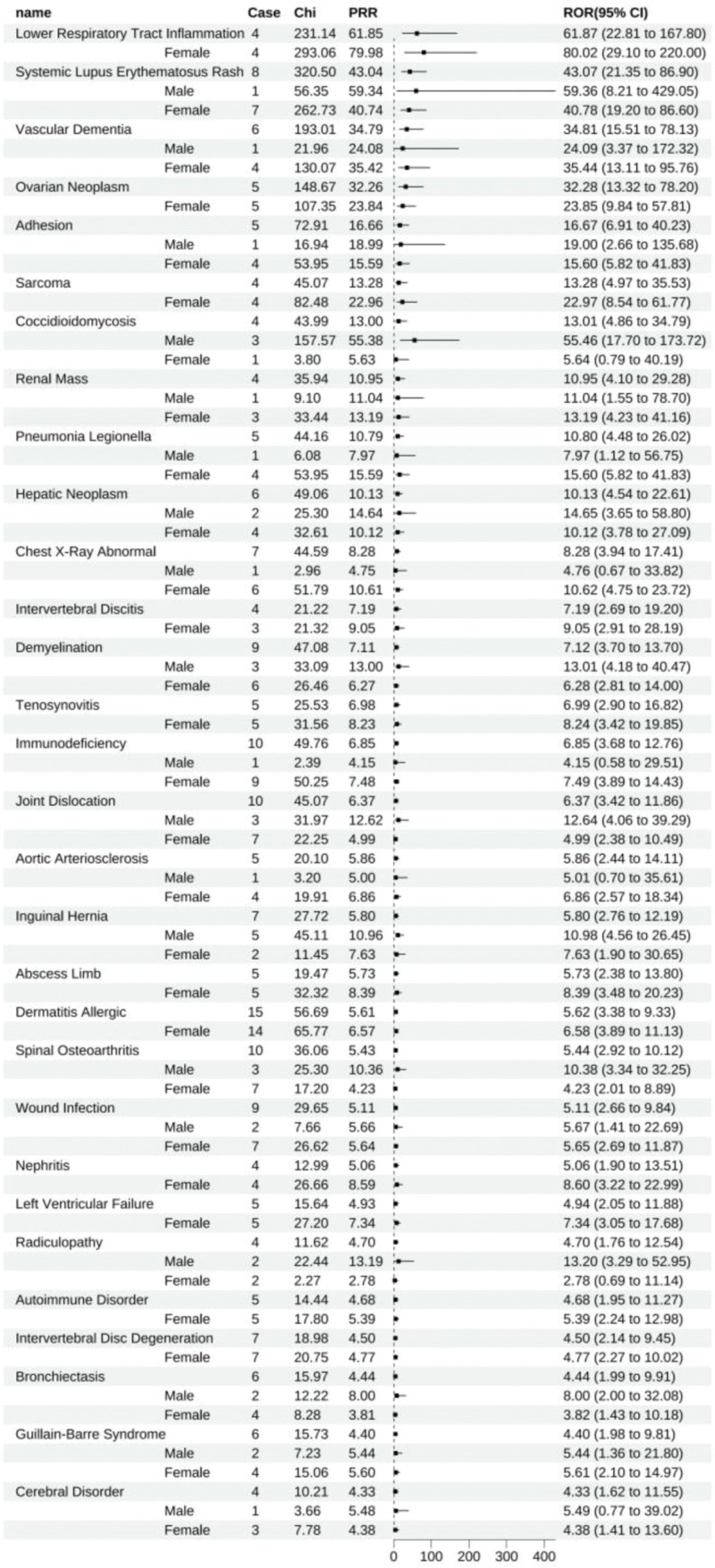
Positive ADE unique to adalimumab. ADE = adverse drug event.

Adalimumab exhibited 63 unique positive adverse drug events (ADEs), including lower respiratory tract inflammation (All: 61.87 [22.81–167.80], female: 80.02 [29.10–220.00]), systemic lupus erythematosus rash (All: 43.07 [21.35–86.90], male: 59.36 [8.21–429.05], female: 40.78 [19.20–86.60]), vascular dementia (All: 34.81 [15.51–78.13], male: 24.09 [3.37–172.32], female: 35.44 [13.11–95.76]), ovarian neoplasm (All: 32.28 [13.32–78.20], female: 23.85 [9.84–57.81]), adhesions (All: 16.67 [6.91–40.23], male: 19.00 [2.66–135.68], female: 15.60 [5.82–41.83]), sarcoma (All: 13.28 [4.97–35.53], female: 22.97 [8.54–61.77]), coccidioidomycosis (All: 13.01 [4.86–34.79], male: 55.46 [17.70–173.72], female: 5.64 [0.79–40.19]), among others (Fig. 9).

**Figure 10.**
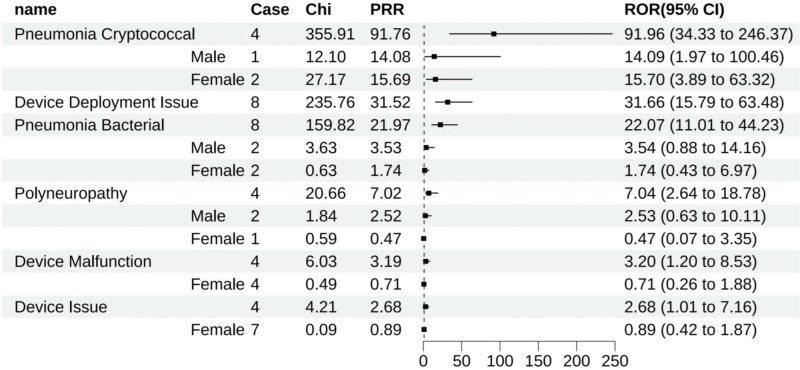
Positive ADE unique to golimumab. ADE = adverse drug event.

Golimumab had 6 unique positive ADEs, which comprised cryptococcal pneumonia (All: 91.96 [34.33–246.37], male: 14.09 [1.97–100.46], female: 15.70 [3.89–63.32]), issues with device deployment (All: 31.66 [15.79–63.48]), bacterial pneumonia (All: 22.07 [11.01–44.23], male: 3.54 [0.88–14.16], female: 1.74 [0.43–6.97]), polyneuropathy (All: 7.04 [2.64–18.78], male: 2.53 [0.63–10.11], female: 0.47 [0.07–3.35]), device malfunction (All: 3.20 [1.20–8.53], female: 0.71 [0.26–1.88]), and other device-related issues (All: 2.68 [1.01–7.16], female: 0.89 [0.42–1.87]) (Fig. 10).

**Figure 11.**
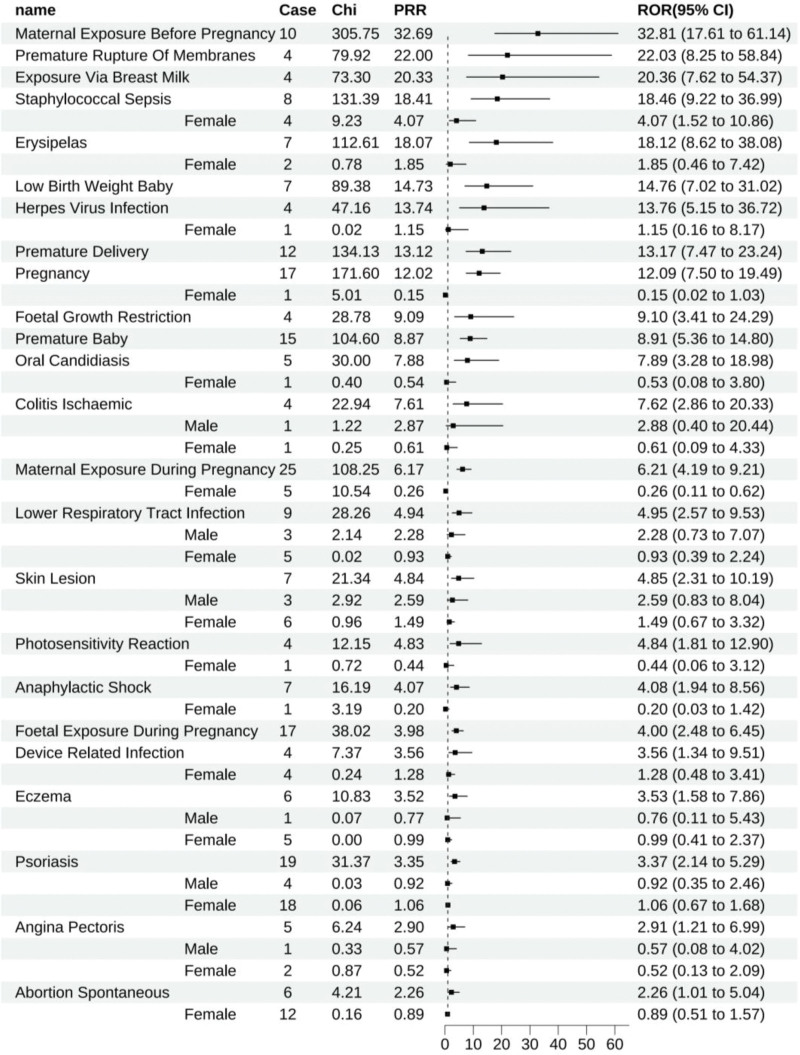
Positive ADE unique to certolizumab. ADE = adverse drug event.

Certolizumab pegol was associated with 24 unique positive ADEs, such as maternal exposure prior to pregnancy (All: 32.81 [17.61–61.14]), premature rupture of membranes (All: 22.03 [8.25–58.84]), exposure through breast milk (All: 20.36 [7.62–54.37]), staphylococcal sepsis (All: 18.46 [9.22–36.99], female: 4.07 [1.52–10.86]), erysipelas (All: 18.12 [8.62–38.08], female: 1.85 [0.46–7.42]), low birth weight in infants (All: 14.76 [7.02–31.02]), herpes virus infection (All: 13.76 [5.15–36.72], female: 1.15 [0.16–8.17]), premature delivery (All: 13.17 [7.47–23.24]), and more (Fig. 11).

**Figure 12.**
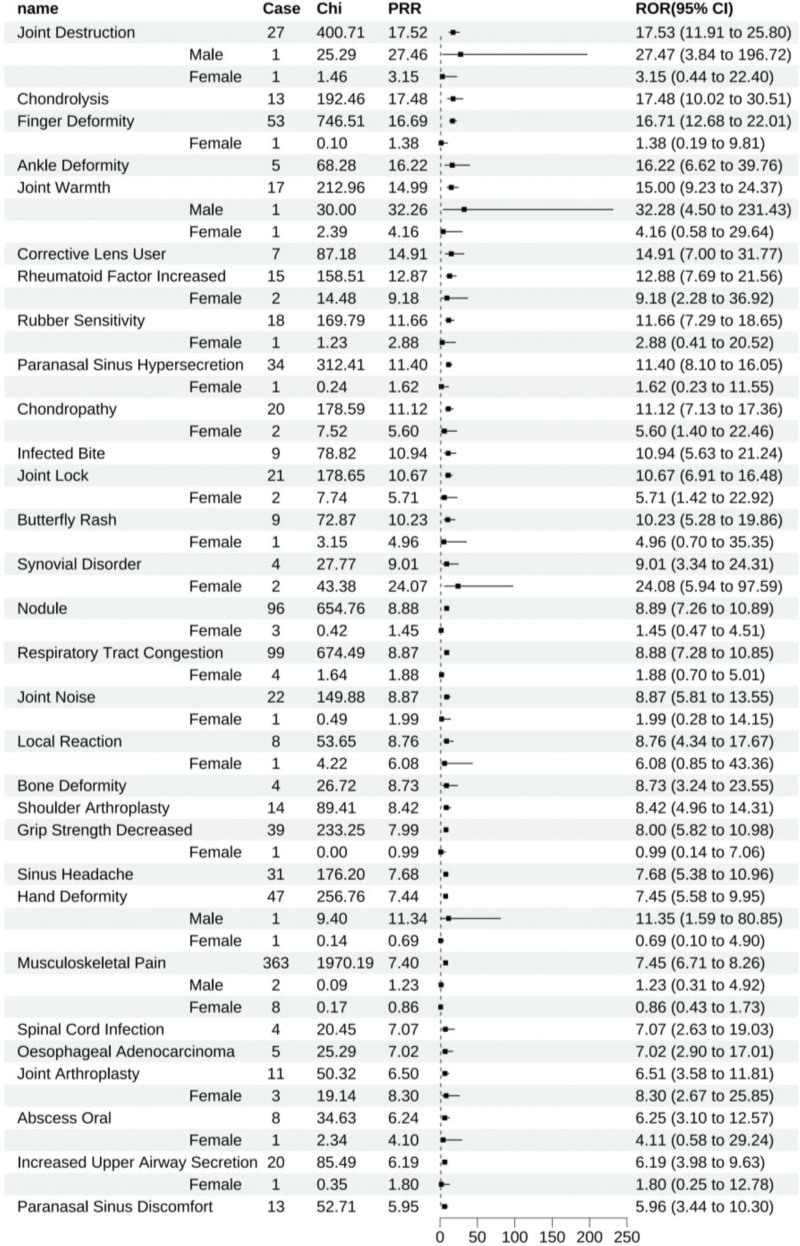
Positive ADE unique to etanercept. ADE = adverse drug event.

Etanercept had 180 unique positive ADEs, including joint destruction (All: 17.53 [11.91–25.80], male: 27.47 [3.84–196.72], female: 3.15 [0.44–22.40]), chondrolysis (All: 17.48 [10.02–30.51]), finger deformity (All: 16.71 [12.68–22.01], female: 1.38 [0.19–9.81]), ankle deformity (All: 16.22 [6.62–39.76]), and joint warmth (All: 15.00 [9.23–24.37], male: 32.28 [4.50–231.43], female: 4.16 [0.58–29.64]), to name a few (Fig. 12).

**Figure 13.**
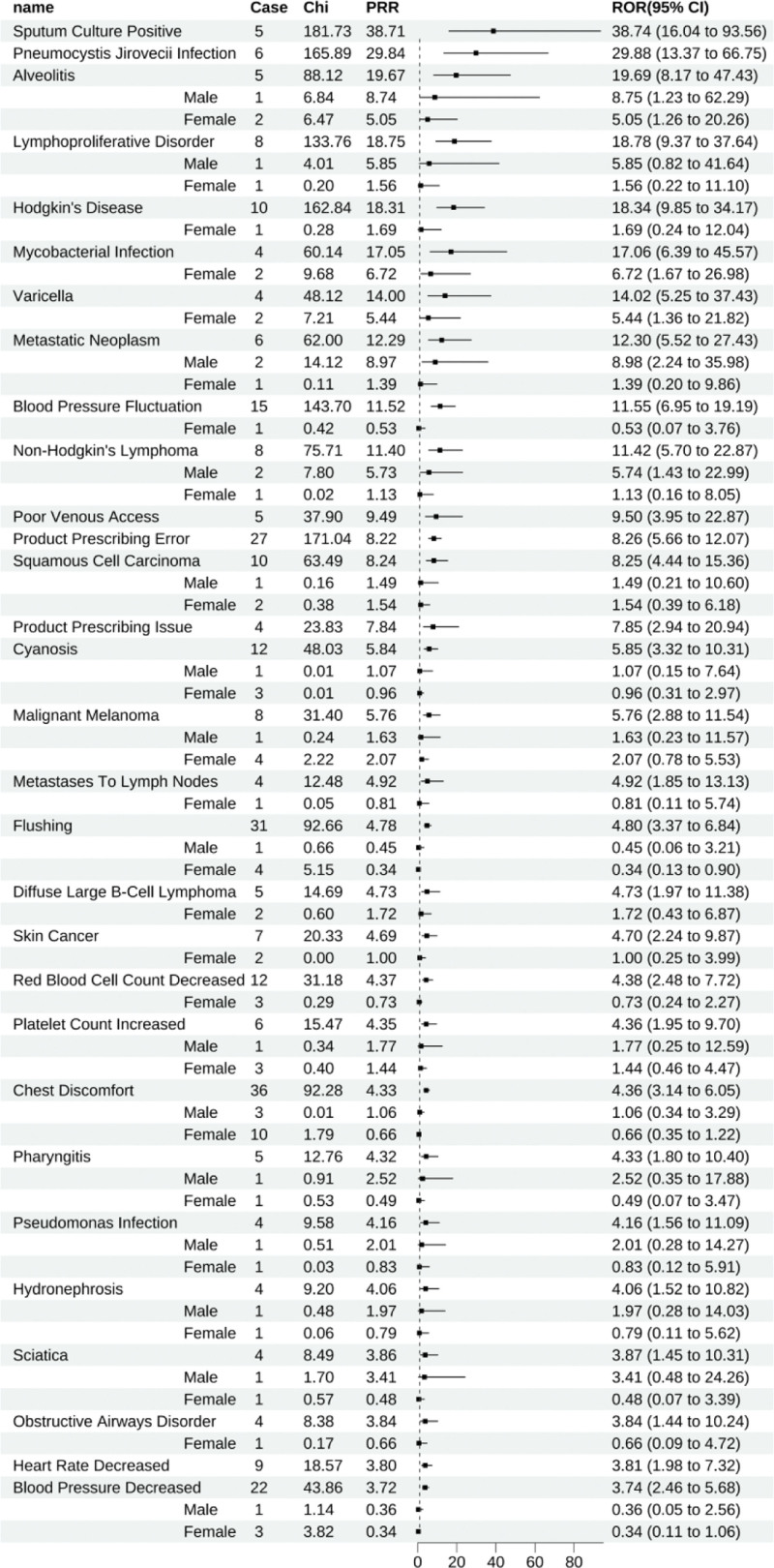
Positive ADE unique to infliximab. ADE = adverse drug event.

Infliximab was linked to 60 unique positive ADEs, with conditions like Hodgkin’s disease (All: 18.34 [9.85–34.17], female: 1.69 [0.24–12.04]), metastatic neoplasm (All: 12.30 [5.52–27.43], male: 8.98 [2.24–35.98], female: 1.39 [0.20–9.86]), non-Hodgkin’s lymphoma (All: 11.42 [5.70–22.87], male: 5.74 [1.43–22.99], female: 1.13 [0.16–8.05]), among the noted (Fig. 13).

## 4. Discussion

Globally, an impressive number of patients have been treated with TNF-α inhibitors such as etanercept, infliximab, and adalimumab, marking a significant advancement in the management of contemporary immunological diseases.

Nevertheless, accurately assessing a drug’s adverse reactions is crucial to ensuring its proper utilization. Regulatory entities and the pharmaceutical industry employ data mining techniques to sift through extensive databases like the FAERS, frequently used to detect AEs potentially induced by new.^[25]^

As the inaugural study to collectively examine the shared and unique adverse drug events (ADEs) among 5 TNFi therapies inpatients with rheumatoid arthritis (RA), the research uncovered significant disparities in hospitalization rates attributable to TNFi treatments, with Golimumabat the highest (47.0%) and Infliximab at the lowest (35.2%). However, all exceeded the substantial threshold of 35.0%, warranting attention. Among the positive signals detected for the medications were 4 ADEs familiar to all 5 TNFis. Under normal conditions, TNF helps modulate immune responses, but an overactive TNF may lead to inflammation and tissue damage.^[26]^ Studies have indicated a correlation between the use of TNFis and an increased risk of herpes zoster occurrences, particularly inpatients suffering from rheumatic diseases such as RA, where TNFi treatment may elevate the risk of contracting herpes zoster.^[27]^

Furthermore, TNF inhibitors may also heighten the risk of non-tuberculous mycobacterial infections.^[28]^ TNFis might augment the likelihood of developing tuberculosis inpatients undergoing treatment for ulcerative colitis, implying an impact on the immune system that increases the vulnerability to pulmonary tuberculosis.^[29]^ However, according to Ziga Rotar,^[30]^ the incidence of tuberculosis inpatients treated with TNFis for conditions such as RA, ankylosing spondylitis, and psoriatic arthritis was commensurate with the prevalence in regions endemic for non-tuberculous mycobacteria, less than one-tenth of these patients required prophylactic treatment. Thus, the real-world mining for ADEs can yield contradictory perspectives. The FAERS database serves as a critical source for detecting real-world signal data, especially for early indicators of potential drug-associated safety issues. The findings of this study highlighted that TNFis could prompt risks of sinusitis, herpes zoster, and tuberculosis, necessitating intensified clinical monitoring for those medicated. The FDA has issued blackbox warnings for adalimumab, golimumab, certolizumab, etanercept, and infliximab, encompassing severe infections such as active tuberculosis and invasive fungal infections, aligning with the results of this study and advising the discontinuation of these drugs should a patient develop a severe infection.

Interestingly, subsequent subgroup analyses of positive ADEs associated with each drug revealed that adalimumab was linked to more detailed infection-related ADEs, such as lower respiratory tract inflammation (ROR = 61.87, 95% CI: 22.81–167.80), coccidioidomycosis (ROR = 13.01, 95% CI: 4.86–34.79), pneumonia legionella (ROR = 10.80, 95% CI: 4.48–26.02), spinal osteoarthritis (ROR = 5.44, 95% CI: 2.92–10.12), and nephritis (ROR = 5.06, 95% CI: 1.90–13.51). Although the FDA’s blackbox warning for adalimumab includes tumorous ADEs, in 52 global clinical trials of adalimumab, aside from lymphoma and non-melanoma skin cancer, the most extended observation of malignant tumors was breast, colon, prostate, lung, and melanoma. However, this study found that ovarian cancer (ROR = 4.09, 95% CI: 1.84–9.13) and liver tumors (ROR = 10.13, 95% CI: 4.54–22.61) could also be potential positive ADEs. In a randomized, non-blinded controlled trial lasting over 2 years conducted by Jobanputra Paresh,^[31]^ 125 RA patients were randomly assigned to receive adalimumab or infliximab, among which 14 serious ADEs occurred, including 2 cases of myocardial infarction fatalities and 1 case of ovarian cancer, consistent with this study’s findings of ovarian cancer, aortic arteriosclerosis (ROR = 5.86, 95% CI: 2.44–14.11), ischaemic cardiomyopathy (ROR = 3.95, 95% CI: 1.48–10.53), and myocardial ischaemia (ROR = 2.68, 95% CI: 1.44–4.99). One study reported that the incidence of biologic-induced autoimmune diseases is estimated at 8 cases per 1000 patients, with a review of cases exposed to different biologics revealing a 0.03% incidence of central nervous system demyelinating diseases.^[32]^ In a recent study, researchers reported that the risk of multiple sclerosis (MS) inpatients with IBD was 1.32 times higher than that in the healthy control group. Additionally, the study found that the incidence of MS in IBD patients exposed to TNFi treatment increased by 43% compared to those not exposed to anti-TNF-α therapy.^[33]^ Despite discontinuation of the drug, three-quarters of patients with TNF-induced demyelination continued to experience neurological issues.^[34]^ Our study discovered that for RA patients, adalimumab potentially induces demyelination (ROR = 7.12, 95% CI: 3.70–13.70) and may increase the likelihood of MS (ROR = 1.90, 95% CI: 1.08–3.36). Although causality cannot be fully confirmed, close monitoring of patients receiving biologic therapy is warranted to diagnose potential neurological complications that may require a change in treatment. No studies have reported a link between adalimumab and vascular dementia (ROR: 34.81, 95% CI: 15.51–78.13); hence, clinical vigilance is required.

Similarly, golimumab has also been reported to potentially cause demyelinating polyneuropathy, with abnormal sensations diminishing after discontinuation of the medication.^[35]^ Our study detected this positive signal as well; however, due to the small number of cases after gender stratification, it was impossible to assess the differences between genders in this context. TNF inhibitors seem to be the safest and most studied biological drugs for use during planned pregnancy, pregnancy, or breastfeeding in the treatment of psoriasis, given their nonexistent or minimal placental transfer. The safest substitute is likely certolizumab,^[36]^ which was approved by the FDA in 2018 for use in pregnant women. Correspondingly, we also detected potential ADEs related to pregnancy and childbirth, including premature rupture of membranes (ROR = 22.03, 95% CI: 8.25–58.84), premature baby (ROR = 8.91, 95% CI: 5.36–14.80), and Low Birth Weight Baby (ROR = 14.76, 95% CI: 7.02–31.02), as well as spontaneous abortion (ROR = 2.26, 95% CI: 1.01–5.04). Notably, we did not detect further serious ADEs, such as teratogenic effects or infant mortality. Research conducted by Corinna Weber-Schoendorfer and colleagues^[37]^ found an increased risk of significant congenital disabilities in the exposed group (5.0%) compared to the non-exposed group (1.5%), as well as an increased risk of prematurity (17.6%), but no increased risk of spontaneous miscarriage (16.2%). The safety of certolizumab for pregnant women is also supported by other studies.^[38–40]^ In our study, certolizumab did not have the fewest positive ADEs. However, due to the restricted use of other TNF inhibitors in pregnant women and the lack of valid report data in this patient group, there is still a need to be cautious about the issue of spontaneous miscarriage.^[41]^ In comparison, this study identified unique positive signals for etanercept about bone diseases such as joint destruction (ROR = 17.53, 95% CI: 11.91–25.80), chondrolysis (ROR = 17.48, 95% CI: 10.02 to 30.51), and finger deformity (ROR = 16.71, 95% CI: 12.68–22.01), which may also be attributed to the progression of RA. RAis a systemic autoimmune disease characterized by persistent synovitis and the destruction of bones and cartilage in multiple joints. Etanercept, a fully human soluble TNF receptor Fc fusion protein,^[42]^ has been proven effective and safe in clinical trials among RA patients.^[43]^ The combined therapy of etanercept and methotrexate has been shown to inhibit the progression of joint destruction and repair bone erosions,^[44,45]^ highlighting its distinct therapeutic benefits against bone damage. To date, no studies have reported the association of etanercept with Joint Destruction ADEs, thus not excluding it as a consequence of disease progression.

Additionally, while infliximab’s positive signals for mycobacterial infection, malignant melanoma, and diffuse large B-Cell lymphoma have been elucidated, our findings also revealed further positive associations: metastatic neoplasm (ROR = 12.30, 95% CI: 5.52–27.43), metastases to lymph nodes (ROR = 4.92, 95% CI: 1.85–13.13), metastases to liver (ROR = 2.72, 95% CI: 1.22–6.06), and metastases to lung (ROR = 2.90, 95% CI: 1.09–7.73). These findings have yet to be previously reported in the literature or product monographs and warrant further clinical attention.

## 5. Conclusion

This study, rooted in a disproportionality analysis of the FAERS database, was designed to uncover potential ADEs associated with TNFi therapies to provide safety evidence for clinical medication use. It recognized that TNF inhibitors share common ADEs, possibly due to impaired immune function, such as herpes zoster, and highlighted the need for careful selection of TNFis, especially in patients with concurrent malignancies, where drugs like Infliximab might be used with greater caution. Despite identifying positive signals within the overall population, the incomplete nature of gender reporting has resulted in less robust findings within gender stratification. At the same time, the ADEs associated with different TNF inhibitors in populations of varying ages, medical histories, and medication histories still warrant further investigation. Additionally, given that case reports are voluntarily submitted and subject to considerable subjectivity, establishing a causal relationship between TNFis and ADEs cannot be conclusively determined.

## Author contributions

**Conceptualization:** Bohui Zheng, Manting Liu, Dongqiang Luo.

**Data curation:** Bohui Zheng, Manting Liu, Dandan Dai, Dongqiang Luo.

**Formal analysis:** Bohui Zheng, Manting Liu, Yifan Shang, Dongqiang Luo.

**Investigation:** Yifan Shang, Xiangyun Dou.

**Software:** Bingshuo Liu, Zilan Zhong.

**Visualization:** Bohui Zheng, Shulan Huang.

**Writing – original draft:** Bohui Zheng, Manting Liu, Dandan Dai, Yifan Shang, Xiangyun Dou, Bingshuo Liu, Zilan Zhong, Shulan Huang.

**Writing – review & editing:** Dongqiang Luo.
